# Interaction between M-Like Protein and Macrophage Thioredoxin Facilitates Antiphagocytosis for *Streptococcus equi* ssp. *zooepidemicus*

**DOI:** 10.1371/journal.pone.0032099

**Published:** 2012-02-27

**Authors:** Zhe Ma, Hui Zhang, Junxi Zheng, Yue Li, Li Yi, Hongjie Fan, Chengping Lu

**Affiliations:** College of Veterinary Medicine, Nanjing Agricultural University, Nanjing, China; University of Padova, Italy

## Abstract

*Streptococcus equi* ssp. *zooepidemicus* (*S. zooepidemicus*, *S.z*) is one of the common pathogens that can cause septicemia, meningitis, and mammitis in domesticated species. M-like protein (SzP) is an important virulence factor of *S. zooepidemicus* and contributes to bacterial infection and antiphagocytosis. The interaction between SzP of *S. zooepidemicus* and porcine thioredoxin (TRX) was identified by the yeast two-hybrid and further confirmed by co-immunoprecipitation. SzP interacted with both reduced and the oxidized forms of TRX without inhibiting TRX activity. Membrane anchored SzP was able to recruit TRX to the surface, which would facilitate the antiphagocytosis of the bacteria. Further experiments revealed that TRX regulated the alternative complement pathway by inhibiting C3 convertase activity and associating with factor H (FH). TRX alone inhibited C3 cleavage and C3a production, and the inhibitory effect was additive when FH was also present. TRX inhibited C3 deposition on the bacterial surface when it was recruited by SzP. These new findings indicated that *S. zooepidemicus* used SzP to recruit TRX and regulated the alternative complement pathways to evade the host immune phagocytosis.

## Introduction

*Streptococcus equi* ssp. *zooepidemicus* (*S. zooepidemicus*, *S.z*), a member of the Lancifield's group C, is an opportunistic pathogen that could infect a wide variety of non-human species, including important domesticated cattle such as horses, cows, swine, sheep, and dogs. In China, *S. zooepidemicus* is the major cause of diseases in swine. Occasionally, it can infect humans via zoonotic transmission from the infected animals and cause invasive infections in humans such as septicemia and meningitis. [1], [2]. Pathogenic microorganisms in a nonimmune host must evade from the innate immune system before the infection can be established [3]. Many pathogens have unique surface structures that can interfere with the phagocytosis by the neutrophils [4]. The hyaluronic acid capsules expressed by many strains can also hinder the phagocytosis process [5]. M protein is an important virulence factor of group A *streptococci*: this fibrillar, surface-exposed protein deters opsonization of the organism using the alternate complement pathway [6], [7]. Previous studies have demonstrated that *S. zooepidemicus* carry antigens with antiphagocytic characteristics similar to the M proteins expressed from Lancefield group A and G *streptococci*; hence it was named M-like protein (SzP) [8]. SzP is a cell surface-anchored protein that confers phagocytosis resistance [9]. SzP-knockout strains had 1000-fold decrease in LD50 compared to the wild type [10]. However, the molecular mechanism by which SzP protects *S. zooepidemicus* from phagocytosis is poorly understood.

Thioredoxin (TRX) is a small multifunctional protein with a redox-active dithiol/disulfide in the conserved active site. The functions of TRX are to reduce protein disulfide bonds and to scavenge hydrogen peroxide together with peroxiredoxins [11]. It was originally identified as a cytokine-like factor in virus-transformed cells [12]. TRX is localized in the cytosol and on the cell surface. The release of TRX in various cells types can be triggered by different extracellular stimuli [13]. Many pathogenic bacteria can evade the complement-mediated host defense by recruiting factor H (FH) to the bacterial surfaces. [14]–[16]. M and M-like proteins show affinity for FH, their interaction is proposed as the mechanism by which M and M-like proteins exert their antiphagocytic effects [17], [18]. The C3 convertase activity analysis found that TRX could inhibit the conversion of C3 to C3a and C3b. This suggested that TRX could have a similar function with FH, which was consistent with previous findings [19].

Our studies were focused on the molecular mechanism(s) by which SzP protects *S. zooepidemicus* from phagocytosis. We identified the SzP/TRX interaction and found that the activity of TRX was not inhibited by this interaction. We also found that TRX could facilitate the antiphagocytic process when it was recruited by SzP anchored on the surface of *S. zooepidemicus*. Further experiments showed that TRX regulated the alternative complement pathway via C3 convertase inhibition and FH association. TRX alone could inhibit C3 cleavage and C3a production, and the inhibitory effect was additive with FH. TRX was able to inhibit C3 deposition on the bacterial surface when it was recruited by SzP of *S. zooepidemicus*. To our knowledge, this was the first reported identification of SzP/TRX interaction. It mediated resistance to the phagocytic activity of macrophages. Our findings could contribute to the general understanding on how SzP confers phagocytosis resistance.

## Results

### *S. zooepidemicus* M-like protein (SzP) interacts with Thioredoxin

**Figure 1:**
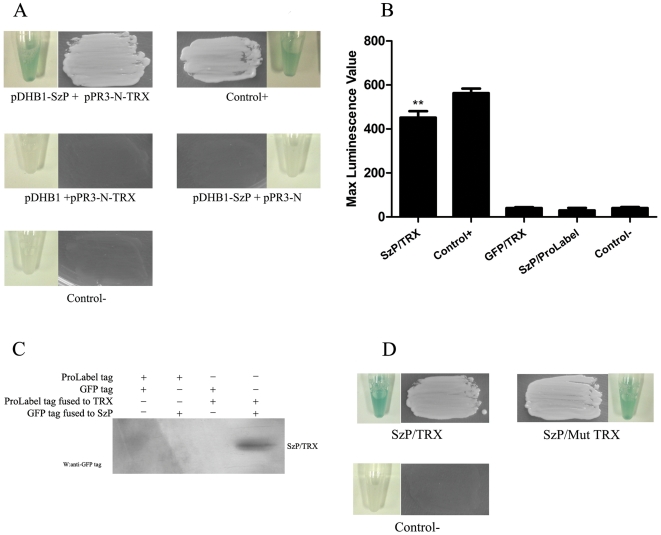
The interaction between SzP and TRX. A: The split-ubiquitin yeast two hybrid assay. SzP was cloned in frame into the yeast expression vector (pDHB1), fused at its N-terminus to a small membrane anchor (the yeast ER protein Ost4) and at its C-terminus to a reporter cassette composed of the C-terminal half of ubiquitin (Cub) and a transcription factor (LexA-VP16). The recombinant TRX-pPR3-N (fusion to the mutated N-terminal half of ubiquitin) and SzP-pDHB1 were co-transformed into the NMY51 yeast strain. The co-transformed cells were selected on Trp-/Ade-/His-/Leu- 80 mM Aminotriazole dropout plates. The control+ represented co-transformation of pDSL-Delta-p53 and pDHB1-largeT (Dualsystems Biotech, Switzerland). Tubes showed the results of Liquid β-galactosidase assay. B: Luminescence value of the substrate corresponding to the pProLabel tag fused to TRX. Histogram showed max luminescence value through 1 h monitoring (n = 3, mean±SD, ** indicates that a value was significantly different (p<0.01)from the control- group). C: SzP was immunoprecipitated from HEK293 cells expressing TRX. HEK293 lysates were immunoprecipitated with rabbit polyclonal antibodies against TRX. The immunoprecipitates were subjected to the western blotting analysis with GFP monoclonal antibodies against GFP -SzP. D: pDHB1-SzP and pPR3-N-mut-TRX were co-transformed into the yeast strain NMY51. SzP interacted with mutant TRX, which had mutations Cys^32^ and Cys^35^ to Ser (C32S/C35S) in its active site. Tubes showed the results of Liquid β-galactosidase assay.

The coding region of ATCC35246 SzP in *S. zooepidemicus* was screened against a porcine pulmonary alveolar macrophage (PAM) cDNA library using the Split-ubiquitin yeast two-hybrid (Y2H) technique. In the Y2H screen, 28 proteins were identified to have a potential interaction with the SzP protein in the yeast cells growing on Trp^−^ Ade^−^ His^−^ Leu^−^ 80 mM aminotriazole media (Sigma). Thioredoxin (TRX, GenBank No. NM_214313.1) was repetitively identified 13 times (Table 1). The fusion plasmids carrying these 28 proteins were isolated after the first round of the screen and retested in fresh yeast cells containing pDHB1-SzP. Only 12 potential SzP interacting proteins were identified in the retest and they were pursued further. Figure 1A showed the interaction between SzP and TRX with appropriate controls. A total of 12 candidate SzP interacting proteins were identified from the retest and their putative functions were listed in Table 1.

**Table 1:** Candidate SzP interacting proteins and their putative functions.

Interactor	Number of hits	Protein function
Thioredoxin	13	In our article
Glia maturation factor gamma-like	5	Maybe involved in glial differentiation, neural regeneration, and inhibition of tumor cell proliferation
Mitochondrial import receptor subunit TOM6 homolog	3	Modulate the assembly and dissociation of the multisubunit preprotein translocase (Tom) machinery of the mitochondrial outer membrane
Selenoprotein K	3	Boosting the immune function in host defence and inflammatory diseases
Protein Sec61 subunit beta-like	2	Proteins enter the ER by the Sec61 translocon, a proteinaceous channel composed of three subunits, alpha, beta and gamma.
BCL2/adenovirus E1B 19 kDa interacting protein 3-like	1	Cause cell death by targeting mitochondria
Syntaxin 8	1	Involved in vesicular trafficking and docking
Transmembrane protein CD9	1	Member of the tetraspan transmembrane protein family, which facilitates, the infection of tissue culture cells with CDV
Guanine nucleotide binding protein (G protein), gamma 11	1	G proteins are heterotrimers consisting of α, β, γ subunits, involved in signal transduction
TRAF3-interacting JNK-activating modulator-like	1	An adapter molecule that specifically regulates TRAF3-mediated JNK activation
Mast cell-expressed membrane protein 1-like	1	Homologous to human granule membrane protein 17
Clone:AMP010010B04, expressed in alveolar macrophage	1	Unknown

The result obtained with the two-hybrid system was confirmed by Co-IP through two independent detection approaches. A ProLabel tag (an enzyme capable of producing a strong chemiluminescent signal via catalyzing its substrate) was genetically integrated at the N-terminal of each of these 12 candidate proteins using the vector pProLabel-C. pProLabel-C or pProLabel-C fused with the candidate protein were then transfected into HEK 293 cells with plasmid pAcGFP1-C, expressing GFP alone, or with plasmid pAcGFP1-SzP, a derivative of pAcGFP1-C coding for a GFP-SzP fusion protein. For the chemiluminescent detection approach, protein G plus/A agarose beads and anti-GFP monoclonal antibodies were incubated with the transfected HEK293 lysate and the luminescence level of the substrate-ProLabel reaction was measured. As seen in Figure 1B, the luminescence signal was only detected when SzP and TRX were both present, while GFP or ProLabel-tag alone was unable to bind with TRX or SzP. TRX was the only one of these 12 candidate proteins that co-immunoprecipitated with SzP *in vitro*. In addition, Western-Blot detection approach was performed. Protein G plus/A agarose beads and anti-TRX antibodies were incubated with the transfected HEK293 lysate and GFP-SzP was detected with the anti-GFP monoclonal antibodies. Figure 1C was consistent with these results. Altogether, we identified the interaction of SzP/TRX for the first time.

### SzP interacts with oxidized and reduced TRX in vitro

**Figure 2:**
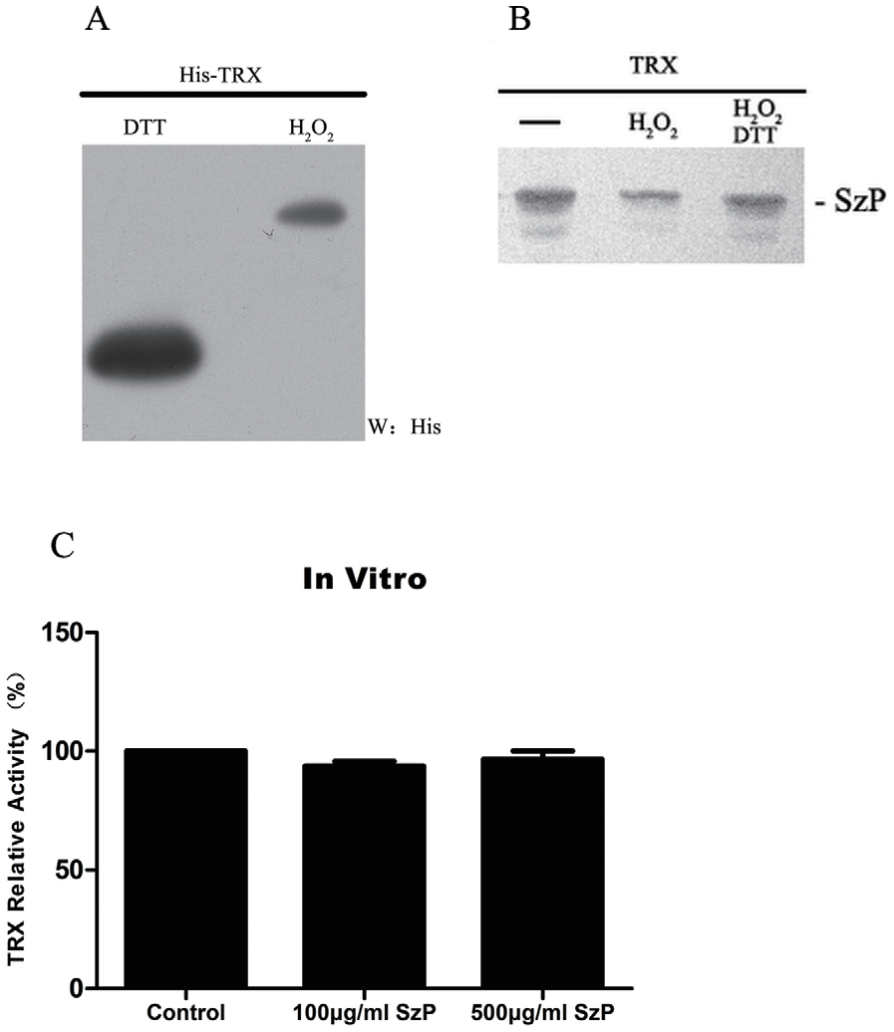
Oxidized and reduced forms of TRX can interact with SzP. A: TRX bound to protein G beads was treated with either 1 mM H_2_O_2_ or 100 mM DTT for 15 min at room temperature. The proteins were separated by native PAGE and visualized by the western blot analysis for His-tag fused TRX. B: Oxidized and reduced forms of TRX were able to interact with SzP. His-TRX bound to protein G beads were incubated with Nonidet P-40 buffer or with buffer supplemented with 1 mM H_2_O_2_ for 15 min at room temperature. After extensive washing in the same buffer, the proteins were submitted to the binding assay. As a control to revert oxidation, a part of the H_2_O_2_-treated fusion protein was incubated with buffer containing 100 mM DTT for 15 min prior to SzP incubation. C: SzP did not inhibit TRX activity. The insulin disulfide reduction assay was performed in the lysates obtained from confluent cultures described under “Materials and Methods.” The activity of TRX bound to SzP *in vitro*: TRX activity was detected after incubating 10 µg TRX with 100 µg/ml or 500 µg/ml SzP at 37°C for 2 h. Control samples were treated with PBS. (n = 3, mean±SD).

We investigated if the SzP/TRX interaction was dependent on oxidized or reduced form of TRX. For this purpose, TRX bound protein G beads were coated with anti-TRX polyclonal antibodies and treated with H_2_O_2_ before the *in vitro* binding assay. We found a clear difference of migration in polyacrylamide gels between the H_2_O_2_-treated and the DTT-treated TRX (Figure 2A). This result suggested the efficiency of the oxidative modification by H_2_O_2_, which was consistent with previous findings [20]. However, the oxidative treatment did not noticeably hinder the SzP/TRX interaction. The interaction was still present with reduced TRX in the DTT treated samples (Fig. 2B).

### SzP and TRX interaction does not inhibit TRX activity

The conserved active site of TRX has two cysteines that are essential for its redox activity. Previous report showed that the active site was where some TRX interaction partners bind [21]. We decided to investigate if this site was also necessary for the binding of SzP. TRX-pPR3-N and Mut-Trx-pPR3-N (C32S/C35S), a Cys32 and Cys35 mutant, were transformed into the yeast strain NMY51 containing a bait plasmid SzP-pDHB. The transformed yeast colonies grew on Trp^−^ Ade^−^ His^−^ Leu^−^ 80 mM aminotriazole selective media. Both the wild-type and the mutant TRX were able to interact with SzP, suggesting that the integrity of the active site was not necessary for this interaction (Fig. 1D).

To investigate whether the SzP/TRX interaction could inhibit TRX activity, we used an *in vitro* insulin reduction assay to measure the reducing activity of TRX. Our results showed that high concentration of SzP did not inhibit TRX activity (Fig. 2C). The SzP/TRX interaction did not inhibit TRX activity and rendered TRX more prone to the complement pathway regulation and other biological functions.

### *S. zooepidemicus* recruits TRX to the surface and facilitates antiphagocytosis

**Figure 3:**
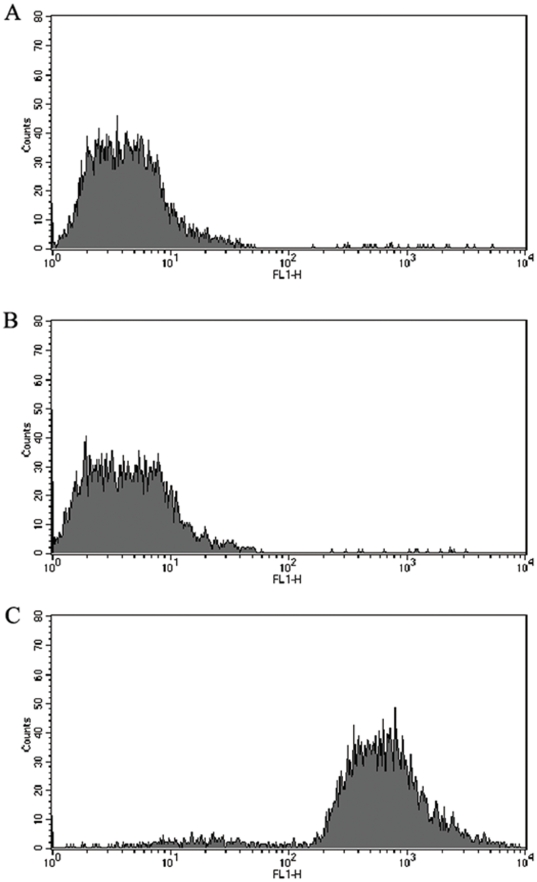
Recruitment of TRX to the surface of viable *S. zooepidemicus.* Flow cytometry results showed that TRX could bind to the surface SzP of *S. zooepidemicus*. A: *S. zooepidemicus* wild strain incubated with PBS (negative control); B: SzP-knockout strain incubated with TRX; C: *S. zooepidemicus* wild strain incubated with TRX. The SzP of *S. zooepidemicus* interacted with TRX and recruited TRX to the bacterial surface.

We further determined that TRX interacted with SzP of viable *S. zooepidemicus*. *S. zooepidemicus* wild strain and the SzP-knockout strain were both incubated with TRX. PBS was used as a negative control. After suitable treatment, all 3 samples were analyzed using flow cytometry. We found that the *S. zooepidemicus* wild type, but not the SzP-knockout strain, was able to recruit TRX to its surface (Fig. 3). This suggested that the surface anchored SzP was important for TRX recruitment.

**Figure 4:**
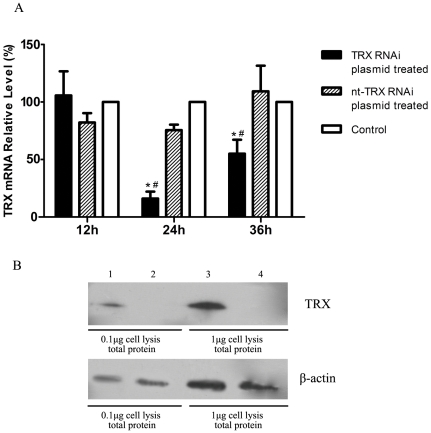
TRX knockdown. A: The relative level of TRX mRNA was detected by RT-PCR. β-actin was used as the reference control. The mRNA level of control was considered as 100%. The relative level of TRX mRNA was calculated using equation 2^−ΔΔCT^ (n = 3, mean±SD, the symbols, * and #, indicate that a value differed significantly (p<0.05) from the control and nt-TRX RNAi treated groups, respectively.); B: Reduction in TRX protein levels was confirmed by the western blot analysis in Raw264.7 cell. Lane 1 and 3 were nt-TRX RNAi plasmid treated cell lysis, lane 2 and 4 were TRX RNAi plasmid treated cell lysis. All samples were extracted at 24 h after transfection.

The recruitment of TRX to the cell surface of *S. zooepidemicus*'s suggested that this interaction could enhance the bacterial adaptation in hosts, thus contribute to the antiphagocytosis of *S. zooepidemicus*. We investigated this issue by assessing the effect of the SzP/TRX interaction on the phagocytosis *in vitro*. We employed RNA interference to knock down endogenous TRX expression in the macrophages. Using RT-PCR, we found that the level of TRX mRNA was reduced to less than 15% in macrophages treated with RNAi than the nt-RNAi control cells (Fig. 4A). We also detected a significant decrease in TRX protein level in RNAi treated cells then the nt-RNAi control cells (Fig. 4B, top panel). The level of β-actin in macrophages was used as an internal control (Fig. 4B, bottom panel).

**Figure 5:**
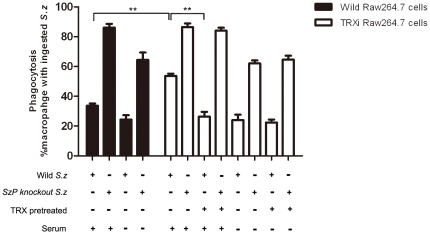
The SzP/TRX interaction facilitated *S. zooepidemicus* to avoid being phagocytized. The phagocytosis percentage of Raw264.7 wild strain and TRXi strain did not differ significantly. The *S. zooepidemicus* SzP-knockout strain were phagocytized effectually. In the presence of the serum, the antiphagocytosis of *S. zooepidemicus* was more pronounced with the SzP/TRX interaction, as there was significantly more macrophage containing ingested *S. zooepidemicus* in TRXi Raw264.7 cells than the wild type Raw264.7 cells. Thus, the SzP/TRX interaction reduced the phagocytosis of *S. zooepidemicus* by the macrophages in the host immune system. The phagocytosis percentage of the TRX pretreated *S. zooepidemicus* wild strain was significantly lower than the SzP-knockout strain in the TRXi Raw264.7 cells. In the absence of the serum, the phagocytosis of the macrophages was independent of TRX, and only SzP was able affect phagocytosis (n = 3, mean±SD, ** p<0.01).

*S. zooepidemicus* wild strain and SzP-knockout strain were tested in an *in vitro* phagocytosis assay Briefly, mouse macrophages with or without mouse serum were treated with the wild strain and TRXi strain cells. The ingestion of SzP-knockout strain in the wild and TRXi Raw264.7 cells suggested that the phagocytosis of the macrophages was not inhibited by the TRX interference. Serum was used as a source of the complement system. It had an opsonophagocytosis effect which allowed the macrophages to ingest bacteria more efficiently. Our result suggested that TRX could hinder the opsonophagocytosis process, as *S. zooepidemicus* containing surface TRX was less ingested by macrophages than the SzP knock-out strain or the non-TRX treated *S. zooepidemicus*. However, the phagocytosis was not affected by TRX in the absence of the serum. SzP facilitated *S. zooepidemicus* to avoid phagocytosis with or without the serum. All these results indicated that TRX could hinder phagocytosis when it was recruited by SzP on the bacterial surface in the presence of the serum. On the other hand, SzP was able to elicit antiphagocytosis responses through another mechanism in the absence of the serum (Fig. 5).

### The complement pathway regulation

**Figure 6:**
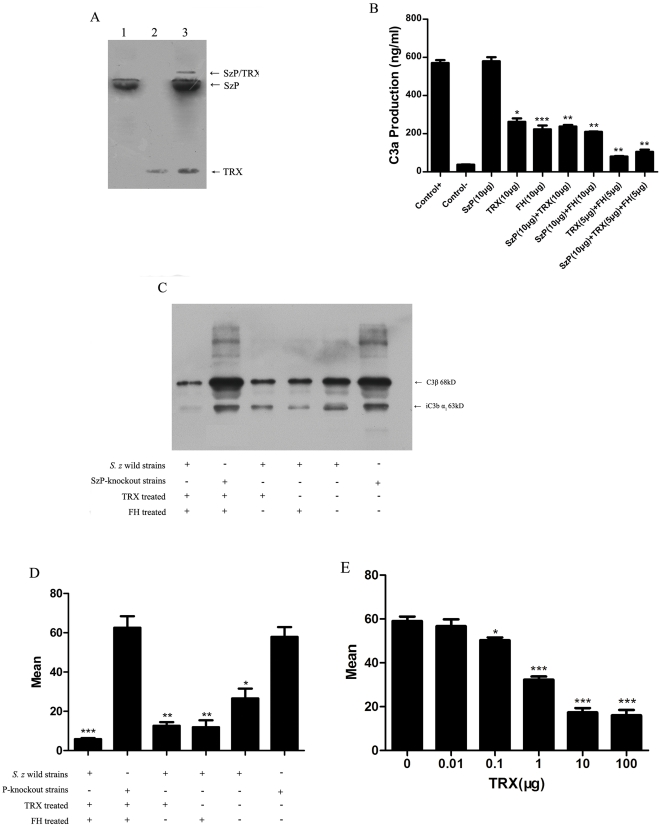
SzP/TRX interaction contributed to the FH recruitment and reduced the C3 deposition on the bacterial surface, beneficial for *S. zooepidemicus* to evade phagocytosis of the host immune system. A: The binding of FH to SzP (lane 1), TRX (lane 2) and the SzP/TRX complex (Lane 3) was demonstrated by using polyclonal goat antiserum to FH in an immunoblot analysis. B: TRX inhibited C3 convertase activity in the fluid phase. Fluid phase alternative pathway C3 convertase was generated by the addition of purified C3, C3i, factor B, and 0.1 M MgCl_2_. 10 µg of TRX or FH were added followed by the addition of factor D to a final volume of 125 µl. Purified components only (control+); purified complement components without factor D (control-). The inhibition of C3 convertase was determined by C3a generation after 30 min of incubation and measured by a C3a ELISA. The effect of dosage increase of FH and TRX on C3a generation (ng/ml) was shown here. Reduction in C3a generation was correlated with the decreased C3 convertase activity. (Symbol * indicates that a value significantly differs from the control+ group). C: Immunoblot showing C3 components eluted from the surface of the *S. zooepidemicus* wild strains and the SzP knockout strains following treatment of TRX, FH or TRX and FH in porcine plasma. The blot was developed with the affinity purified antiserum to C3. C3 components eluted from the *S. zooepidemicus* wild strains and the SzP knockout strains after incubation in porcine plasma were used as the negative control. D: Flow cytometry analysis of *S. zooepidemicus* surface-bound C3b, a total of 10,000 events were collected per sample and a single gate was used to exclude debris. E: Flow cytometry analysis of gradient concentration TRX pretreated *S. zooepidemicus* surface-bound C3b. The antiphagocytic effectiveness of the TRX was dose-dependent until saturation. These results were the mean±SD for n = 3, *p<0.05, **p<0.01, ***p<0.001.

We used the immunoblotting analysis to demonstrate that FH interacted with SzP, TRX and SzP/TRX complex. Previous report found that TRX interacted strongly with FH [19]. We found that SzP of *S. zooepidemicus* had affinity with FH as well. We also found that the SzP/TRX complex, visualized by the band above SzP and TRX, was able to bind with FH (Fig. 6A).

The effect of TRX, FH and SzP on the activity of C3 convertase in the alternative complement pathway was determined using the fluid phase assay. Next, we examined the effect of TRX on FH, a major regulator of the alternative complement pathway and an inhibitor of the C3 conversion to C3a and C3b. Using the fluid-phase technique, the production of C3a from C3 conversion was detected by adding factor B and factor D (Fig. 6B). We found that TRX alone inhibited C3 cleavage. Furthermore, we found that the inhibitory effect was additive with FH, as 5 µg TRX and 5 µg FH together had a stronger inhibition on the activity of C3 convertase than 10 µg TRX or 10 µg FH alone. SzP alone did not show any effect on C3 cleavage. These results indicated that SzP could recruit TRX as a regulator of the alternative complement pathway and the regulation of TRX could also be distinct from the regulation of FH.

Phagocytes can recognize foreign particles most efficiently after opsonization with serum-derived opsonins such as IgG and C3b or inactive C3b. Studies using C3 antibodies (anti-C3) showed that TRX and FH both prevented C3b deposition on the *S. zooepidemicus* wild strain (Fig. 6C, 6D). We detected more C3b deposition on the cell surface of SzP-knockout strain compared to the *S. zooepidemicus* wild strain after treatment with TRX and FH. Immunoblotting with C3 specific antiserum revealed that the level of surface localized C3b and iC3b, the major derivatives of C3, was reduced after TRX or FH treatment in the presence of SzP. The antiphagocytic effectiveness of the TRX was dose-dependent, but when bacteria was saturated by TRX, the antiphagocytosis stopped increasing. (Fig. 6E)

## Discussion

There are generally cross-talks between bacteria and the host immune system. *S. zooepidemicus* has developed a multitude of strategies to evade host defenses by interfering many aspects of the immune system. Such interference can normally lead to the down-regulation or inhibition of the inflammatory responses. Previous studies have indicated that SzP in *S. zooepidemicus* shares similar antiphagocytic characteristics with the M proteins in the Lancefield groups A and G streptococci [22]. SzP of *S. zooepidemicus* is a 40.1-kD surface-anchored protein that elicits serum opsonic and protective responses in mice and horses [9]. We cloned SzP gene of *S. zooepidemicus* ATCC35246 and investigated the mechanism by which they conferred antiphagocytic effects against the host immune responses.

Initial attempt to identify SzP interaction partners using the classical yeast two-hybrid system was not successful. Classical Y2H requires both proteins to interact in the nucleus and the nuclear environment may cause certain proteins to fold improperly and thus abolish their interactions with other proteins [23]. Next, we used the split-ubiquitin yeast two-hybrid system to detect SzP interaction partners. This system does not require protein interactions to occur in the nucleus and is therefore ideal for cytosolic proteins as well as membrane proteins. The C-terminal of SzP is anchored to the membrane while the rest of the protein is highly hydrophilic. We conjectured that the SzP/TRX interaction occurred at the cytosolic side of the plasma membrane.

The current study identified the interaction between SzP and TRX. We further provided three lines of evidence to verify that SzP was associated with TRX; (i) An interaction between SzP and TRX was screened and confirmed in the split-ubiquitin yeast two-hybrid system. (ii) SzP and TRX were co-immunoprecipitated from pAcGFP1-SzP and pProLabel-TRX transfected HEK293 cells, and the result was detected with ProLabel-tag enzyme activity and confirmed with western-blot. (iii) FH was able to bind with SzP and TRX, as well as the SzP/TRX complex. This suggested that the binding of SzP to TRX was biologically relevant.

TRX plays a variety of redox-related roles that are conserved from *E. coli* to humans. It is a small, ubiquitous, multifunctional protein that contains a redox-active disulfide/dithiol within its active site, -Cys-Gly-Pro-Cys- [20]. TRX mutants with Cys32/Cys35 to Ser mutations were still able to interact with SzP (Fig. 1D). Furthermore, both oxidized and reduced forms of TRX were able to interact with SzP (Fig. 2B). This suggested that the redox active site was not the site of SzP/TRX interaction. It was found that oxidization leads to TRX dimerization through a disulfide bond between Cys73 in both monomers [24], [25]. Therefore, the residues necessary for SzP interaction were not hindered in the dimeric form of TRX. It was interesting to note that the activity of TRX was not inhibited by SzP *in vitro*. These results could explain why SzP/TRX interaction did not inhibit TRX activity.

To better understand how surface protein SzP could contribute to the pathogenecity of *S. zooepidemicus*, we investigated the interaction of *S. zooepidemicus* with Raw264.7 cells, using the wild strain ATCC35246 (which is virulent to mice) and SzP-knockout strain attenuated in virulence. We employed flow cytometry to detect the recruitment of TRX to the surface of viable *S. zooepidemicus*. This recruitment was mediated by SzP, since SzP-knockout strain was unable to recruit TRX to the surface. We also found that both the *S. zooepidemicus* wild type and the SzP-knockout strain were efficiently phagocytized by macrophages when TRX was absent, though the wild strain was harder to be ingested than the SzP-knockout strain. We believed that there should be another mechanism of SzP to elicit antiphagocytosis responses in *S. zooepidemicus*. The current work provided evidence that the *S. zooepidemicus* wild strain could avoid being phagocytized much more effectively, whereas the mutant strains were rapidly ingested by Raw264.7 cells even in the presence of TRX. All these results were obtained in the presence of the serum. However, TRX could not facilitate antiphagocytosis responses in the absence of the serum, it was only related to SzP. These results indicated the expression of SzP allowed the bacteria to recruit TRX, which had effects on the complement pathway. Therefore, the SzP/TRX interaction facilitated the antiphgocytic response of the *S. zooepidemicus*

Previous report found that the wild-type *S. pyogenes* expressing M protein and/or M-like proteins on the cell surface could survive inside the neutrophils [18]. *S. pyogenes* mutant strains that lacked either M protein and/or M-like proteins were rapidly killed. M and M-like proteins display affinity for several human plasma proteins such as IgG [26], C4 BP [27], fibrinogen [28] and FH [29]. It may be possible that these interactions could interfere with normal host immune mechanisms, including phagocytosis. We believed that SzP in *S. zooepidemicus* elicit antiphagocytosis through its interaction with TRX.

FH can inhibit the conversion of C3 to C3a and C3b and inactivate C3b. It is recognized as the main regulator of C3 convertase. Many pathogenic organisms evade phagocytosis by coating their surface with the host FH [30]. We asked if *S. zooepidemicus* could evade phagocytosis by a similar method via SzP/TRX interaction. Our results showed that SzP, TRX and SzP/TRX complex were able to bind with FH. This suggested that the SzP/TRX interaction did not prohibit *S. zooepidemicus* to recruit FH on its surface. We reasoned that although SzP itself could recruit FH to the cell surface, SzP/TRX interaction was still important because TRX could act as a regulator of the alternative complement pathway not only in association with FH but also on its own. TRX acted additively to FH in the alternative complement pathway. The increased level of the capsules may enhance phagocytosis resistance, but could also reduce adhesion to the mucosal surface [31], [32]. has less hyaluronic acid than *S. equi*. Therefore, *S. zooepidemicus* has stronger adhesiveness but lower level of capsules, which rendered it more susceptible to phagocytosis [33]. Having an effective antiphagocytosis mechanism is therefore essential to *S. zooepidemicus*. Recruiting FH via SzP and TRX to its cell surface would certainly contribute to phagocytosis evasion. When FH was not abundant, *S. zooepidemicus* could use TRX as succedaneum of FH. C3 deposition experiments showed that TRX and FH both prevented deposition of C3b on *S. zooepidemicus*, which was pretreated with both TRX and FH limited deposition of opsonic C3b on the bacterial surface. This indicated that the SzP/TRX interaction contributed to the antiphagocytosis response in *S. zooepidemicus* via the inhibition of the C3b deposition.

In conclusion, the SzP/TRX interaction was a novel antiphagocytic mechanism of *S. zooepidemicus*. The SzP/TRX interaction did not influence the TRX activity and function. It also contributed to the FH recruitment and reduced the C3 deposition on the bacterial surface, allowing *S. zooepidemicus* to be more evasive to the alternative complement pathways. These mechanisms were very beneficial for *S. zooepidemicus* to evade phagocytosis of the host immune system. Additional studies of this interaction will undoubtedly help us to understand better how SzP is involved in the antiphagocytosis mechanisms.

## Materials and Methods

### Ethics

Porcine PAM was obtained with consent from one healthy pig under the ethical approval granted by the Nanjing Agricultural University Veterinary College. The protocol was approved by the Science and Technology Agency of Jiangsu Province. The approval ID is SYXK (SU) 2010-0005. All efforts were made to minimize animal's suffering.

### Cell culture, bacterial strains and transfection conditions

Raw264.7 cells (ATCC) and HEK 293 cells (ATCC) were maintained in the Dulbecco's modified Eagle medium (DMEM) high glucose (Gibco, Invitrogen Corp., Carlsbad, CA) supplemented with 10% fetal bovine serum (FBS). Cells were used from passages 5–20.

The *S*. *Zooepidemicus* wild type strain ATCC35246 and the *S. zooepidemicus* SzP-knockout strain was constructed by our lab [10]. *Escherichia coli* strains DH5α, DH10B and BL21, *Saccharomyces cerevisiae* strain NMY51 (MATa his3Δ200 trp1-901 leu2-3,112 ade2 LYS2::(lexAop)_4_-HIS3 ura3::(lexAop)_8_-lacZ ade2::(lexAop)_8_-ADE2 GAL4) were used in this study. *S*. *zooepidemicus* was cultured with fresh Todd-Hewitt broth (THB) medium. *E.coli* was cultured with fresh Luria–Bertani (LB) medium. *S. cerevisiae* was cultured with fresh Yeast Extract Peptone Dextrose Adenine hemisulfate (YPDA) medium.

Raw264.7 cells and HEK 293 cells were transiently transfected using Lipofectamine 2000 and opti-MEM (Invitrogen Corp., Carlsbad, CA) according to the manufacturer's instruction. Media were changed 6 h post transfection and the cells were treated or assayed 24 h post transfection. *S. cerevisiae* was transformed using the LiAc method [34]. Lysates were subject to Western-blot analysis to confirm the expression of the bait gene in the pDHB1 plasmid.

### Split-ubiquitin yeast two-hybrid assay

We used the Split-ubiquitin yeast two-hybrid DUALhunter system (Dualsystems Biotech, Switzerland) to identify SzP interaction partners from the porcine macrophages [35], [36]. The coding region of *S. zooepidemicus* ATCC35246 SzP (GenBank No. EU624402.1), excluding the signal sequence, was amplified by PCR using primers 5′- GCGGCACGGCCATTACGGCCGTTGAGTCAGCTAAGCCTGTA -3′(SfiI) and 5′- GCAGCGCGGCCGAGGCGGCCTTTTCTTTGCGTCTTGTTGAC -3′(SfiI). PCR product was then inserted into the Split-ubiquitin yeast two-hybrid Cub domain vector pDHB1 to generate the ‘bait’ plasmid pDHB1-SzP, which was verified by DNA sequencing. The expression of the bait protein was confirmed. pDHB1-SzP was used to screen a porcine PAM cDNA library (constructed by our lab) for identifying SzP interacting proteins. Positive yeast clones containing the library plasmid encoding Szp interacting proteins were purified and retested for their growth phenotypes. Plasmid DNA preparations for these yeast clones were generated by the Yeast Plasmid Extraction Kit (Biomega). The insert fragment of these prey plasmids was detected by PCR amplification using primers pPR3N-F (5′- GTCGAAAATTCAAGACAAGG -3′) and pPR3N -R (5′- AGCGTGACATAACTAATTAC - 3′). The chosen prey plasmids were amplified in DH5α, recovered through ampicillin selection and identified by DNA sequencing with pPR3N-F primer. The cDNA sequences were used to search GenBank and NCBI BLAST against the porcine genome. After banishing duplication, the remaining plasmids were re-transformed into the yeast cells containing pDHB1-SzP. Liquid β-galactosidase assay was used to retest for their interaction with SzP in the yeast again.

### Co-Immunoprecipitation assay

GFP was fused at the N-terminal of SzP in the *S. zooepidemicus* strain ATCC35246 using the vector pAcGFP1-C (Clontech, TAKARA Bio, USA). ProLabel tag was fuse at the N-terminal of 12 porcine candidate proteins using the vector pProLabel-C (Clontech). These 12 plasmids were each co-transfected with pAcGFP1-SzP into HEK 293 cells and cultured on 100 mm plates. Cells were harvested after 36 h incubation and lysed with the lysis buffer containing 20 mM Tris, 200 mM NaCl, 1 mM EDTA, 0.5 NP-40 and Protease inhibitor Cocktail (Pierce, USA) and 1× PMSF (Sigma, USA). Cell lysates were incubated 2 h with anti-GFP monoclonal antibody (Beyotime) at 4°C. Next, 30 µl of protein G plus/A agarose beads (Clontech) was added and incubated at 4°C overnight. Next day, the beads were washed five times with the lysis buffer and the activity of ProLabel was measured using the ProLabel Detection Kit II (Clontech). pAcGFP1-p53 and pProLabel-T were used as the positive control, while pAcGFP1-Lam and pProLabel-T (Clontech) were used as the negative control.

After the initial identification of the Szp/TRX interaction, it was again confirmed with Co-IP and western-blot. Protein G plus/A agarose beads together with anti-mouse TRX polyclonal antibodies (Proteintech, USA) were incubated with HEK293 cell lysates. The samples were washed extensively in the lysis buffer. The beads were boiled in the sample buffer, and the supernatants were loaded onto SDS-PAGE gels. To confirm the SzP/TRX interaction, western-blot against GFP-SzP was performed using anti-GFP monoclonal antibodies.

### Reducing/Oxidizing Reagent Treatment

Oxidization treatment for TRX was performed following a previously publish protocol [37]. Briefly, protein G beads coated with anti-TRX polyclonal antibodies were bound to TRX and treated with 1 mM H_2_O_2_ in Nonidet P-40 buffer for 15 min and then washed 6 times with the buffer prior to SzP incubation. As a control to revert oxidation, a part of the H_2_O_2_-treated fusion protein was incubated with buffer containing 100 mM DTT for 15 min prior to SzP incubation. After 2 h incubation, samples were centrifuged and washed five times with Nonidet P-40 buffer. The precipitated proteins were subjected to SDS-polyacrylamide gel electrophoresis and detected by western-blot using anti-SzP monoclonal antibodies [38].

### Mutagenesis and TRX activity assay

We used “QuikChange II Site-directed Mutagenesis Kit” to construct Cys^32^ and Cys^35^ to Ser (C32S/C35S) double mutations in TRX using the TRX-pPR3-N vector as the template (Stratagene). Primers were 5′ CTCAGCCACGTGGTCTGGGCCTTCCAAAATGATCAAGCC 3′ and 5′ GGCTTGATCATTTTGGAAGGCCCAGACCACGTGGCTGAG 3′. The mutant plasmid was named mut-TRX-pPR3-N, and transformed into the yeast strain containing SzP-pDHB1 plasmid to detect the interaction between SzP and mutant TRX.

TRX activity assay was performed in 96-well plates with an end point insulin assay [39], [40]. We incubated 10 µg TRX with 100 µg/ml or 500 µg/ml SzP at 37°C for 2 h. Samples were mixed with 3.8 µl 1.7 mM TRX reductase from rat liver (Sigma), 20 µl 1∶12 mixture of solution N(40 mg/ml β-NADPH) ,solution M(210 mM HEPES, pH 7.6, 790 µM insulin, 20 mM EDTA) and TE (50 mM Tris-HCl, pH 7.6, 20 mM EDTA) to reach a total volume of 50 µl. The reaction mixture without TRX reductase was used as the control for background readings. After incubating in 37°C for 30 min, 200 µl of 1 mM 5, 5′- dithiobis (nitrobenzoic acid) in 6 M guanidine hydrochloride solution was added to stop the reaction and the absorbance was measured at 412 nm.

### Recruitment of TRX to the surface of viable *S. zooepidemicus*

The flow cytometry analysis of *S. zooepidemicus* ATCC 35246 cultured *in vitro* was performed as described previously [41]. Briefly, 5×10^7^ CFU/mL wild strain or the SzP knockout was incubated for 30 min at 37°C in 2 mg/mL TRX. The bacteria cells were washed with phosphate-buffered saline (PBS; pH 7.2), collected by centrifugation, and incubated with rabbit anti-mouse TRX polyclonal antibodies (Proteintech, USA) for 30 min at 37°C. This was followed by incubation with FITC-conjugated goat anti-rabbit IgG secondary antibodies (Beyotime, China). Surface-bound TRX was fluorescently stained and analyzed by a FACS Calibur (Becton Dickinson). Data were compared by the analysis of variance with Tukey's correction for multiple comparisons and Student's t-test. P values<0.05 were considered significant.

### Macrophage TRX knockdown and Phagocytosis assays

siRNA templates were designed using online siRNA Target Finder (Ambion, Japan) to match 20 nonconserved nucleotide sequences within the mouse TRX mRNA. The oligo nucleotide sequences used to create pBAsi-mU6-TRX were iTRX-sense 5′- GATCCGGCTTGAAGCCTCTATTACTTTCAAGAGAAGTAATAGAGGCTTCAAGC TTTTTTACGCGTG -3′ and iTRX-antisense 5′-TCGACACGCGTAAAAAAGCTTGAAGCCT CTATTACTTCTCTTGAAAGTAATAGAGGCTTCAAGCCG -3′. The oligonucleotides were annealed and cloned into pBAsi-mU6 (TAKARA Bio, Japan). The expression of TRX siRNA was regulated by the mU6 promoter. Cells were harvested 24 h and 36 h after pBAsi-mU6-TRX transfection. The lysates were collected for western-blot analysis and total RNA was extracted to confirm TRX knockdown using RT-PCR analysis.

The phagocytosis assay was performed using a previously described method with some modifications [42]. The *S. zooepidemicus* wild type strain was cultured to mid-exponential phase of growth (optical density of 0.35–0.45 at 600 nm), washed, and resuspended in Dulbecco's phosphate-buffered saline at 1.25×10^8^ CFU/ml. Bacteria cells were labeled with 5.0 µg/ml FITC (Sigma) for 20 min at 37°C and the unbound labels were removed by 2 washes in DPBS. Labeled *S. zooepidemicus* were resuspended in DMEM and chilled on ice. The Raw264.7 cells (10^6^) or TRX knock-down cells (10^6^) were combined with 10^7^ *S. zooepidemicus* wild type or the SzP knock-out strain in a 96-well microtiter plate on ice. Samples were mixed gently with a pipette and rotated at 37°C for 60 min. At the desired times, samples were placed on ice and analyzed by flow cytometry. Samples were analyzed to determine the total number of Raw264.7 cells with bound/ingested bacteria and then re-analyzed immediately in the presence of an equal volume of Trypan Blue (2 mg/ml in 0.15 M NaCl/0.02 M citrate buffer, pH 4.4) to measure the number of Raw264.7 cells with ingested bacteria (phagocytosis). A total of 10,000 events were collected per sample and a single gate was used to exclude debris and free bacteria (Cell Quest Pro Software, BD Biosciences). Phagocytosis percentage was determined by the percentage of FITC positive Raw264.7 cells after quenching with Trypan Blue.

### FH binding assay

Binding of SzP and TRX to FH was determined using the method previously described with some modifications [43]. SzP, TRX and SzP/TRX complex were incubated at 37°C for 2 h before being subjected to SDS-PAGE and electro-transferred to a PVDF membrane. After blocking with 5% non-fat dry milk in TBST buffer, the membrane was incubated with fresh porcine 1∶200 serum dilution followed by 1∶8 goat anti-human FH serum dilution (Calbiochem, La Jolla, CA) in TBST. After washing in TBST, the blot was developed with HRP conjugated rabbit anti-goat IgG antibodies and followed by Chemiluminescence with SuperSignal® West Pico Substrate (Pierce, USA).

### C3 Convertase Measurement

C3 convertase activity was measured as previously described with some modifications [44]. Briefly, the C3 convertase was assembled in PBS by the addition of the following purified complement components: 10 µg C3 (Sigma); 50 ng C3i; 2 µg factor B (Calbiochem); and 12 µl 0.1 M MgCl_2_. C3i, also known as C3(H_2_O), was generated by five freeze/thaw cycles of the purified C3. Varying concentrations of TRX, SzP, and the SzP/TRX complex were added with or without 10 µg FH, followed by 200 ng factor D (Calbiochem). The reaction mixture was incubated at 37°C for 30 minutes. The generation of C3a was measured by ELISA (Quidel, San Diego, CA). All experiments included positive controls (C3, C3i, factor B, and factor D) and negative controls (C3, C3i, and factor B).

### C3 binding assay

The C3 deposition assay was performed using a previously reported protocol with some modifications [45]. Aliquots of *S. zooepidemicus* wild type and SzP-knockout strains (1.5×10^8^ cells in 150 µl of THB medium) were incubated with 10 µg TRX, 10 µg FH or TRX+FH for 30 min. Fresh porcine plasma (180 µl) incubated at 37°C was then added and mixed with shaking. Complement deposition was stopped immediately after 30 min by the addition of 12 µl 0.5 M EDTA (pH 8.0).

Flow cytometry analysis: The cells were spun down by centrifugation at 13,000 g for 2 min. Cell pellets were washed twice and resuspended in PBS. Surface-bound C3b was detected with anti-C3 antibodies (Sigma) and FITC-labeled goat anti-rabbit IgG (Beyotime). The binding of the antibodies was measured by flow cytometry. A total of 10,000 events were collected per sample and a single gate was used to exclude debris (Cell Quest Pro Software, BD Biosciences).

Western-blot analysis: the cells were spun down and resuspended in 80 µl 1 M hydroxylamine in carbonate buffer (pH 9.0) after washing with PBS-1% SDS twice. The mixture was incubated at 37°C for 1 h with gentle shaking. After centrifugation, aliquots (25 µl) of the supernatant were subjected to SDS-PAGE and transferred to a PVDF membrane. The membrane was incubated with a 1∶100 dilution of anti-C3 antibodies (Sigma), and developed using HRP conjugated goat anti- rabbit IgG antibodies (Beyotime, China). The bands were visualized by Chemiluminescence with SuperSignal® West Pico Substrate (Pierce, USA).

### Statistical analysis

All statistics were performed using an unpaired two-tailed *t*-test with a 95% confidence interval. In all experiments, error bars were denoted using the standard deviation (±SD) of the samples in triplicates or more.
