# Correction: Interaction between M-Like Protein and Macrophage Thioredoxin Facilitates Antiphagocytosis for *Streptococcus equi* ssp. *zooepidemicus*

**DOI:** 10.1371/annotation/a1d856e3-285c-4728-b817-c030fb5ec20b

**Published:** 2012-05-18

**Authors:** Zhe Ma, Hui Zhang, Junxi Zheng, Yue Li, Li Yi, Hongjie Fan, Chengping Lu

Funding statement should read "This study was supported by Program from the National Basic Research Program (973) of China (2012CB518804), the National Natural Science Foundation of China (31172319), the key Technology R?D Program of Jiangsu (BE2009388), the Fundamental Research Funds for the Central Universities (KYT 201003), and the Project Funded by the Priority Academic Program Development of Jiangsu Higher Education Institutions. The funders had no role in study design, data collection and analysis, decision to publish, or preparation of the manuscript."

